# Building a single market for data: Data spaces as pro-competitive infrastructure

**DOI:** 10.1016/j.dib.2026.112839

**Published:** 2026-05-12

**Authors:** Effimia Styliani Konstantopoulou

**Affiliations:** KU Leuven, Center for IT and IP Law, Leuven, Belgium

**Keywords:** Data spaces, Competition law, Data governance, Federated infrastructure, Market design, EU internal market, Data sovereignty

## Abstract

European data spaces constitute pro-competitive infrastructures deliberately designed to align with EU competition law objectives. Unlike proprietary ecosystems that risk entrenching market dominance, data spaces embed neutrality, openness, and non-discrimination into their architectural design, thereby addressing market contestability through governance frameworks rather than ex post regulatory intervention. The main argument advances that data spaces represent a transformative "soft-market intervention" that shifts competitive dynamics from exclusive data hoarding to innovation-based rivalry on shared datasets.

The paper demonstrates how data spaces extend the principle of free movement to data as a potential "fifth freedom" within the EU internal market, while their federated governance structure prevents any single participant from monopolizing data access or determining participation terms. Through ex ante pro-competitive design, data spaces reconcile the need for large-scale data aggregation with competition law safeguards, enabling economies of scale and scope without creating dominance risks. The technical infrastructure emphasizes interoperability by design, federated cloud architecture, and open standards that prevent vendor lock-in and foreclosure effects.

By providing structured alternatives to proprietary data marketplaces controlled by dominant gatekeepers, European data spaces demonstrate how institutional architecture can embed pro-competitive safeguards into market design itself, while fostering digital sovereignty and sustainable competitive advantage within the EU's evolving data economy.

## 1 Introduction

The EU’s policy goal to build a single market for data rests on the premise that data is both the ‘lifeblood of economic development’ and a key factor of production, that is essential to scale innovation and competitiveness.^1^ The European Strategy for Data identifies data spaces as the infrastructures through which this vision can be realised, as they are federated, interoperable, and trustworthy infrastructures that enable large-scale data use, while safeguarding sovereignty and fairness.^2^ As will be argued in this paper, safeguarding competition law is integral to their design. Unlike proprietary ecosystems that risk entrenching dominance, data spaces are conceived as governance frameworks that respect, by design, the provisions of EU competition law by embedding neutrality, openness, and non-discrimination into the rules of participation.^3^ Thus, European data spaces represent a transformative approach to building a sovereign, secure, and interoperable data economy that will define Europe's digital future through 2030 and beyond.

The necessity for such infrastructure stems from a fundamental problem in the contemporary digital economy which is the structural tendency toward data concentration. Currently, the data economy is dominated by proprietary ecosystems where a few gatekeepers extract, hoard, and control vast datasets, creating insurmountable barriers to entry for smaller actors and stifling downstream innovation. Alternative regulatory models such as ex post antitrust enforcement (e.g., Article 102 TFEU abuse of dominance cases) or mandatory utility-style data access regimes, often prove procedurally sluggish or overly coercive, failing to provide real-time, dynamic data access. Therefore, the narrative of this paper positions European data spaces as a structural alternative to the status quo: an ex ante market design that corrects the failures of proprietary ecosystems by replacing data hoarding with governed, interoperable data sharing.

This paper advances the claim that data spaces constitute pro-competitive infrastructures, deliberately structured to align with the objectives of EU competition law. Their architecture addresses issues of market contestability not through compulsory access or direct regulatory intervention, but rather by embedding compliance and interoperability into the terms of participation.^4^ In this sense, data spaces represent what is described in academic literature as an “ecosystem approach” to the data economy, where federated governance and shared standards ensure that the benefits of data aggregation are not captured by a single gatekeeper.^5^

As the Commission has emphasised, “The infrastructures should support the creation of European data pools enabling Big Data analytics and machine learning, in a manner compliant with data protection legislation and competition law, allowing the emergence of data-driven ecosystems.”^6^ In this sense, data spaces can be understood as soft-market interventions, as they are voluntary but rule-based frameworks that embed contestability and transparency into the data economy, complementing traditional competition law enforcement by shaping markets before structural risks arise.

However, the pro-competitive benefits of such infrastructures should not be taken for granted. The very mechanism of large-scale data aggregation introduces inherent antitrust vulnerabilities. Unregulated data sharing and standard-setting risk facilitating informational collusion under Article 101 TFEU and particularly through tacit algorithmic coordination and risk generating unintended dominance effects or infrastructural bottlenecks under Article 102 TFEU.

The argument proceeds in three steps. First, data spaces are situated within the EU’s broader internal market, showing how they extend the principle of free movement to data as a potential “fifth freedom.”^7^ It is within this context that the paper will critically examine the aforementioned antitrust risks inherent in data-driven collaboration. Second, the paper analyses the structural features of data spaces that are aligned with competition law safeguards and elaborates on their ex ante pro-competitive design as a necessary mitigation mechanism. Finally, the paper examines the pro-competitive characteristics of data spaces across three categories: *(a) technical infrastructure, (b) governance model and (c) economic dynamics.*

## 2 Framing Data Spaces

### 2.1 Data spaces as a policy and legal concept

Data spaces emerged as a central concept in the European Data Strategy launched by the European Commission, positioning data as a powerful asset for the public sector, businesses, and individuals to harness the full potential of the digital economy.^8^ Across Europe, vast amounts of data are being collected every second. The development of a robust European Data Spaces ecosystem aims to promote access and the availability of data for economic and societal use, while maintaining data sovereignty and trust for the originating entities.^9^ Although the Data Governance Act (DGA) does not provide a formal legal definition of “data space,” it embeds the concept within its recital framework, stating that the European Strategy for Data envisioned a common European data space as a single market for data that transcends physical storage locations.^10^ This policy context supports the broader conceptualisation of data spaces in academic literature and especially the DSSC’s definition, which describes a data space as a distributed system, underpinned by governance structures, that facilitates secure, trustworthy, and sovereign data transactions.^11^

### 2.2 Data spaces as structured governance systems

According to the definition and the policy conception of a European Data Space, data spaces represent more than just a technical platform. They are structured, distributed systems grounded in robust governance frameworks and infrastructures that facilitate secure, trustworthy, and sovereign data sharing among participants.^12^ At its core, the governance framework establishes clear roles, responsibilities, and rules that regulate how the data space operates and is managed.^13^ Data transactions within this system go beyond simple data exchange, since they require coordinated legal, financial, technical, and organizational mechanisms to ensure that data sharing is both effective and compliant.^14^ These transactions are enabled by a comprehensive data space infrastructure, which includes all the necessary components such as technical systems, legal agreements, and procedural protocols that sustain and support the operational integrity of the data space.^15^ In practice, this entails the role of a federator, who provides key enabling services such as catalogues and brokers for data discovery, mechanisms for identity management and trust establishment, and frameworks for usage control.^16^ These services allow data to remain at its original source while still being shared under enforceable policies, thereby ensuring sovereignty and compliance. Crucially, this approach prevents any single organization from assuming the role of a central gatekeeper, supporting a more open and balanced data ecosystem.

Using the example of Gaia-X, which acts as a federated infrastructure initiative, one can see how shared services, such as identity and trust management, federated catalogues, and data exchange compliance, are designed to ensure that data is not centralised and no single participant controls the essential levers of access.^17^ This minimises the risk of gatekeeping while fostering interoperability across ecosystems.

### 2.3 Synergies between multiple data spaces

Most importantly, the ultimate benefit of data spaces is the coexistence of multiple data spaces and the synergies generated between them. The gained efficiency, increased impact or other benefits of two or more data spaces functioning together, are greater than if the data spaces were working separately, since functionalities are offered by data integration.^18^ The synergies between data spaces can be enabled by common practices, communication concepts, services and components, which increase data space interoperability and enable harmonized processes of using different data spaces.^19^

The benefits of the synergies are illustrated by a concrete case study of two EU data spaces working together, conducted by the Centre of Excellence for Data Sharing & Cloud (CoE-DSC), that involves the Smart Connected Supplier Network (SCSN) and Catena-X.^20^ The collaboration between the SCSN and Catena-X exemplifies the practical integration of EU data spaces to generate synergies that surpass the benefits of operating individually. SCSN, primarily oriented towards manufacturing companies in the high-tech sector, facilitates the sharing of order and product data along supply chains,^21^ while Catena-X, developed under the German automotive industry’s leadership, focuses on enabling secure data sharing for automotive production with an emphasis on traceability and sustainability.^22^ Their integration addresses overlapping challenges such as complexity in supply chain coordination and the need for transparency, enabling participants from both sectors to benefit from interoperable data connectors and harmonized governance frameworks that respect data sovereignty and regulatory compliance. This interoperability not only streamlines processes such as production scheduling and material recycling but also expands business opportunities by allowing automotive suppliers to leverage SCSN’s strengths in order process management. Challenges such as user onboarding, trust establishment through digital certificates, and aligning differing business models are addressed through common standards and trust anchors. This collaboration between SCSN and Catena-X illustrates how federated data space models can foster cross-sector innovation, ecosystem scale, and operational efficiency, situating the Netherlands as a key player in the European data space landscape and demonstrating the wider EU strategy for digital sovereignty and connected data ecosystems.^23^

### 2.4 Towards a single market for data

The core objectives of data spaces revolve around enabling secure, sovereign, and value-generating data sharing among multiple participants. They aim to foster interoperability and collaboration while ensuring regulatory and ethical compliance. In doing so, data spaces are designed to uphold key principles such as data sovereignty, a level playing field, decentralised infrastructure, and public–private governance.^24^ Their ultimate goal is to enhance innovation through the respectful use of data, thereby maximising societal benefit and leveraging the transformative potential of the digital economy.

These objectives directly underpin the broader European vision of creating a single market for data. The vision of the European Data strategy is to create a single market for data that will boost Europe’s global competitiveness, facilitate data flow across sectors, and make available high-quality data for creation and innovation.^25^ This policy goal fully aligns with the vision of data spaces that are built on prima facie openness, interoperability and trust, characteristics that support competition dynamics.

Whereas sector specific data spaces are tailored to each field and address the different needs and challenges of specific areas such as industry, health, agriculture, finance, mobility, education, energy and public administration, a Common European Data Space^26^ would merge the synergies of all the sector specific data spaces and promote innovation and data-driven transformation across Europe. The overarching objective is to facilitate the seamless integration of data within the internal market, enabling its flow with the same efficiency and interoperability as that of physical goods within supply chains.^27^

## 3 Data Spaces as Competitive Catalysts

### 3.1 Free movement of data in the internal market

At the backbone of the European Union’s internal market lie the Treaty of the Functioning of the European Union freedoms, notably the free movement of goods, services, persons and capital enshrined in Articles 26 and 28–66 TFEU.^28^ In recent policy discourse, data has increasingly been framed as a potential *fifth freedom*^29^, whose free flow across Member States is essential to completing the internal market in the digital age.^30^ If data is recognised as a fundamental good within the internal market, then the facilitation of its cross-border movement becomes as indispensable to Union competitiveness as the circulation of goods and services, and must equally be facilitated.^31^

The EU’s data-driven economy is built upon this principle, promoting the free movement of both personal and non-personal data as essential to unlocking innovation, economic growth, and societal benefits.^32^ Yet to realise this freedom in practice requires infrastructures that guarantee circulation under conditions of openness, trust, and interoperability. This is core function of European data spaces.

### 3.2 Antitrust risks in data-driven collaboration

While data spaces are conceptualised as pro-competitive infrastructures designed to dismantle proprietary data silos, the very mechanism of large-scale, structured data aggregation inevitably triggers profound scrutiny under European competition law.^33^ The fundamental tension lies in the fact that while interoperability and free circulation rectify market failures, they simultaneously and drastically alter market transparency. Under Article 101 of the Treaty on the Functioning of the European Union (TFEU), the systematic exchange of commercially sensitive information, whether raw, processed or manipulated data, such as production capacities, pricing strategies, or consumption patterns, can readily facilitate collusive outcomes.^34^ As the European Commission expressly elucidates in its 2023 Horizontal Guidelines, such exchanges artificially reduce strategic uncertainty, thereby enabling undertakings to predict competitors' behaviour and coordinate market conduct without the need for explicit cartel agreements.^35^ The Commission warns that sharing disaggregated, forward-looking, or real-time data is particularly likely to generate conditions of competition that depart from normal market dynamics, effectively replacing the imperative of independent economic determination with tacit coordination.^36^

This risk of tacit coordination is highly acute in dynamic, real-time data infrastructures, such as the envisaged European Mobility Data Space (EMDS). Recent scholarship cautions that the continuous, mandated exchange of granular, temporal data such as dynamic fares, routing logistics, and seat availability, undermines the antitrust doctrine of independent economic conduct.^37^ By establishing unprecedented market transparency, these frameworks inadvertently provide the architecture for ‘digital collusion’ or ‘collusion by code’.^38^

In digital markets where pricing is increasingly delegated to algorithms, the pooling of high-frequency data allows automated systems to monitor market deviations and penalise defection instantaneously. Consequently, data spaces risk functioning as digital conduits that crystallise the internal stability of anti-competitive arrangements, an outcome the Commission explicitly identifies as a primary competition concern in modern data-sharing initiatives.^39^

Beyond pure information exchange, the operationalisation of data spaces necessitates standard-setting agreements and interoperability protocols, which introduce distinct antitrust vulnerabilities rooted in governance asymmetries. If a data space is governed by incumbent market players who possess disproportionate influence over the rule-making process, standardisation can be weaponised. For example if data-sharing schemes involve collective agreements among competitors to determine data access remuneration, they risk classification as price-fixing by object under Article 101(1) TFEU.^40^ Furthermore, the absence of rigid, pro-competitive substantive standards can yield restrictions by effect. Empirical evidence from the implementation of the Second Payment Services Directive (PSD2) demonstrates that allowing incumbents broad discretion over Application Programming Interface (API) parameters often results in technical fragmentation and ‘data gatekeeping’.^41^ This dynamic enables incumbents to manipulate technical standards to raise rivals' costs, ultimately facilitating the anti-competitive foreclosure of small and medium-sized enterprises (SMEs) that lack the resources to absorb the resulting technical friction.^42^

Distinct from the collaborative risks governed by Article 101, the architecture of data spaces also potentially intersects with unilateral conduct governed by Article 102 TFEU. Dominant undertakings may exploit collaborative data structures to entrench their market power or impose unfair trading conditions. Regulatory investigations into digital incumbents, such as the Commission's *Amazon Marketplace* probe and the *Meta* case, illustrate how the forced extraction or unilateral accumulation of third-party data by a dominant firm constitutes an exploitative abuse that leverages informational asymmetries to foreclose nascent competitors.^43^ Within the context of sectoral data spaces, if a shared repository achieves significant market coverage, it may trigger the essential facilities doctrine.^44^ Under this rigorous standard, a dominant undertaking’s refusal to grant access to an indispensable, non-replicable dataset or granting access solely on discriminatory terms could constitute an exclusionary abuse, transforming a theoretical data commons into an infrastructural bottleneck.^45^ Despite these substantial risks, European competition law provides analytical mechanisms to reconcile data collaboration with market contestability. Data pools and federated spaces may escape the prohibition of Article 101(1) or benefit from an individual exemption under Article 101(3) TFEU if robust governance safeguards are embedded ex ante. The Commission acknowledges that efficiencies generated by data sharing, such as enhanced predictive analytics or machine learning training, can outweigh restrictive effects, provided the exchanged data is historical, aggregated, or managed by an independent trustee subject to strict confidentiality.^46^ Furthermore, literature suggests that structuring data spaces as ‘data cooperatives’ with democratic, one-member-one-vote governance, coupled with strict adherence to Fair, Reasonable, and Non-Discriminatory (FRAND) access terms, neutralises the threat of exclusionary standard-setting.^47^

Ultimately, mitigating these antitrust risks requires an institutional design that actively channels data synergies toward innovation, precluding the space from degrading into a vehicle for oligopolistic consolidation.

### 3.3 Data spaces as ex ante market design

#### 3.3.1 Ex ante structural safeguards and federated governance

The defining feature of data spaces is their ex ante pro-competitive design. At the core of their pro-competitive design, data spaces allow for the concentration of data without conferring market power upon any single participant. This structural quality is not incidental but fundamental and is what makes data spaces an institutional tool for shaping markets before anticompetitive risks arise and remedial action becomes necessary. By embedding safeguards of openness, interoperability, and non-discrimination into their very architecture, it is ensured that the benefits of data aggregation are shared among a wide range of actors under legally and ethically sound governance frameworks. Unlike proprietary data ecosystems that entrench dominance through exclusive control, data spaces facilitate access to large, valuable datasets without enabling abuse of concentration.^48^

The organisational model through which this is achieved is the federated and decentralised governance structure.^49^ Data spaces are not centralised repositories but federated ecosystems, where data remains under the control of its holders yet becomes accessible through common interoperability protocols and governance frameworks.^50^ This federated architecture ensures that aggregation does not entail ceding ownership or control but instead, all participants retain sovereignty while benefiting from the scale and scope of shared data. Neutral intermediaries, standardised identity and access management services, and harmonised contractual and technical rules act as ex ante safeguards that prevent any single undertaking from monopolising the data space.^51^

A practical illustration is Catena-X in automotive supply chains. Conceived as the first open and collaborative data space for the sector, Catena-X moves beyond the traditional model of closed OEM-centred networks and extends participation to tens of thousands of suppliers, service providers, and downstream actors.^52^ Its architecture is deliberately designed around federated services for identity management, catalogues, and policy enforcement, ensuring that data remains with its holder while still being discoverable and usable under common rules.^53^ By embedding these neutral services at the governance layer, Catena-X prevents any single manufacturer from monopolising access or determining participation, thereby fostering an ecosystem where collaboration, interoperability, and competition can take place on equal terms.

In addition, in industrial ecosystems it is underscored that the federated nature of data spaces further equally distributes market power across multiple incumbents, by allowing both large incumbents and SMEs to share and use data without reproducing hierarchical dependencies.^54^

By distributing authority and embedding neutrality at the level of governance, data spaces structurally counteract the concentration of market power that typically arises from exclusive control over large datasets.^55^

It has been argued that voluntary data-sharing arrangements and precisely the federated model of European data spaces, can generate significant efficiencies but risk violating Article 101 TFEU through collusion or exclusion if governance structures prove insufficient.^56^ Such collaboratives necessarily exchange competitively sensitive information (e.g., production requirements, customer preferences), creating potential for price coordination unless counteracted by robust safeguards, such as democratic control mechanisms (one member, one vote), interoperable technical standards, and transparent access protocols.^57^ European data spaces operationalise these principles through neutral federators, standardised identity management, and non-discriminatory participation requirements, ensuring shared datasets drive innovation rather than cartelisation.^58^

#### 3.3.2 Preventive nature of market design

The analysis so far has highlighted the preventive nature of the governance model that is used for data spaces. It has been shown that federated data ecosystems can reconcile domain-specific autonomy with system-wide interoperability, thereby avoiding both fragmentation and centralisation.^59^ In competition law terms, this implies that data spaces create a setting in which the benefits of aggregation, such as the economies of scale, scope, and innovation are designed to be realised without tipping the market in favour of one actor. However, ex post competition remedies like mandating collaboration or data sharing are procedurally complex and often fail to deliver real-time access, taking years while data value dissipates.^60^ The ex ante safeguards built into data spaces therefore aim to minimise reliance on such interventions by addressing risks at their source. The fact that design features that can be described as pro-competitive are implemented in the data space by definition, even before any data processing activity takes place, creates a market design environment that by design favours competition law. While it must be critically acknowledged that preventing market dominance does not automatically guarantee consumer welfare, European data spaces are not merely neutral technical pipelines and are rather purpose-driven infrastructures explicitly designed to maximise societal benefits. The ex ante safeguards built into their design actively channel data concentration toward public interest objectives rather than private rent-seeking. For example, the European Health Data Space (EHDS) is legislatively engineered to leverage secondary data use not just for commercial innovation, but to directly improve public health outcomes and policy-making.^61^ Similarly, the Common European Mobility Data Space (EMDS) structures data sharing specifically to reduce transport emissions, optimise shared mobility, and empower sustainable consumer choices.^62^ In the agricultural sector, the Common European Agricultural Data Space ties data pooling to concrete socio-economic and environmental sustainability targets, boosting innovation for farmers and the food supply chain.^63^ Therefore, while the success of these spaces relies on active participation, their foundational governance ensures that if innovation occurs, it is structurally aligned with consumer welfare and broader societal goals.

Therefore, the preventive character of data spaces can be understood as embedding pro-competitive safeguards into the market design itself, even prior to any data processing activities. By structuring participation rules, access conditions, and interoperability mechanisms ex ante, the data space reduces the likelihood that data concentration will evolve into anti-competitive dominance. Instead of assuming guaranteed outcomes, this design makes such concentration more likely to serve as a driver of innovation and consumer welfare, provided governance remains robust.

In contrast, traditional competition law enforcement is reactive and authorities intervene ex post, once an infringement has occurred and market dynamics have already been distorted. Such interventions typically require significant resources and may involve complex remedial measures, including structural remedies, behavioral commitments, or long-term monitoring to restore competitive balance. These measures, while necessary, often entail delays and may not fully reverse the harm suffered by market participants or consumers, as seen in protracted cases like Google Shopping, where leveraging rather than outright refusal frames the abuse but access remains contested.^64^

By comparison, the institutional architecture of data spaces mitigates these risks at their source. Their ex ante pro-competitive design minimizes the need for corrective intervention, thereby reducing both the likelihood and the intensity of competition law infringements. In this respect, data spaces exemplify a preventive governance model that complements traditional competition law by fostering a market environment inherently less prone to unlawful practices.

#### 3.3.3 Future oriented market design

European data spaces are positioned to unlock vast repositories of industrial and commercial data that can be used to train next-generation AI models while respecting confidentiality, competition, and legal boundaries. Through their design data spaces reconcile the need for aggregated, interoperable data that are necessary for the development of Artificial Intelligence applications, digital services, and innovation, with safeguards that prevent competitive harm. This approach offers an alternative to the current reliance on publicly scraped web content by providing access to curated, high-quality corporate data.

The architecture supports cross-sector collaboration through standardized interfaces and governance mechanisms that enable data sharing between traditionally separate domains. This capability is essential for addressing complex challenges such as climate change, healthcare innovation, and smart city development that require integrated data from multiple sectors.

Thus, the concentration of data within these spaces, aligned with legal, ethical, and regulatory standards, becomes a driver of consumer and societal benefit, not a threat to competition. Rather than creating risks of dominance, aggregation in data spaces enhances the free flow of information, sustains market contestability, and advances the overarching objectives of the EU digital single market.^65^ These forward-looking features underscore that data spaces should be conceived not solely as technical infrastructures but as forms of soft-market intervention that regulate access and competition primarily through governance mechanisms rather than coercive measures.

#### 3.3.4 Data spaces as soft-market interventions

Data spaces can be understood as a form of soft-market intervention. Rather than imposing direct sanctions and ex post remedies, data spaces operate as forward-looking infrastructures that proactively shape the conditions for contestable and dynamic digital markets. Their interventionist quality lies in the fact that the rules of participation, the governance model, and the regulatory standards are defined ex ante and embedded in the functioning of the space. Access conditions, interoperability requirements, and safeguards for openness and fairness are thus pre-set, providing a structural framework within which participants operate. At the same time, entry into a data space remains voluntary since undertakings and public bodies are not compelled to join, but those that do participate agree to comply with the common standards. This combination of pre-set governance and voluntary participation illustrates why data spaces are best understood as soft-market interventions, shaping markets through infrastructure and rules, while avoiding coercive or compulsory measures.^66^

This soft-interventionist character becomes particularly evident when data spaces are contrasted with traditional proprietary data marketplaces. Platforms such as AWS data exchange, Microsoft Azure Marketplace, Cloud Public Datasets,^67^ are operated by dominant providers that act as gatekeepers to data. Access in these marketplaces is centralised and determined by unilateral contractual terms, which may include exclusivity clauses, restrictive licensing, or technical lock-in that ties users to the provider’s infrastructure. These conditions privilege incumbents that can absorb costs or negotiate favourable terms, while raising entry barriers for SMEs and start-ups with limited bargaining power. It has been argued that such asymmetries “create barriers for growing a competitive data economy, where all stakeholders can freely participate under fair conditions.”^68^ In competition terms, these structural imbalances risk entrenching dominance and enabling exclusionary conduct of the kind addressed under Article 102 TFEU^69^.

By design, European data spaces function as soft-market interventions aimed at dismantling the barriers created by proprietary data control. Rather than relying on a single gatekeeper, they operate through federated and decentralised governance models in which participation rules, interoperability standards, and fairness safeguards are pre-set and applied equally to all actors.^70^ This means that while entry remains voluntary, participation is conditional on compliance with common standards, which is a mechanism that aligns freedom of choice with structural guarantees of openness. Concrete initiatives such as the European Mobility Data Space (EMDS)^71^ and the European Health Data Space (EHDS)^72^ illustrate this soft-interventionist model: both create shared infrastructures that establish common conditions for accessing critical datasets, thereby enabling SMEs, start-ups, and public institutions to compete with incumbents in delivering innovative services in sustainable mobility and healthcare. In this way, data spaces demonstrate how soft regulatory interventions, grounded in governance and infrastructure rather than coercion, can correct structural imbalances, lower entry barriers, and sustain contestability in digital markets.

#### 3.3.5 Designing data spaces to address scale and fragmentation

The exponential growth in the volume, variety, and velocity of data that reached an estimated 149 zettabytes in 2024 and continues to grow at an exponential rate,^73^ poses challenges that traditional data management frameworks cannot adequately address. Data are generated across highly heterogeneous domains, ranging from structured financial records and sensor data to unstructured social media content, each subject to distinct quality, privacy, and regulatory requirements.^74^ Because data are produced in incompatible formats and under divergent metadata, licensing and compliance regimes, and because their high velocity outpaces the development of common standards, the cost of lawfully reusing them is pushed onto market participants, which fractures the landscape into proprietary silos that only data-rich incumbents can integrate at scale, raising entry barriers and enabling the leverage of market power in competition-law terms.^75^ Left unmanaged, such fragmentation produces interoperability bottlenecks, creates information asymmetries, and entrenches competitive advantages for incumbents able to internalise these costs.^76^ Such fragmentation is not only technical but contractual. More specifically, high transaction costs, incomplete contracts, privacy and security risks, and bargaining asymmetries often discourage voluntary B2B data sharing, producing precisely the type of market failures that competition law seeks to address.^77^

The design of data spaces responds directly to the above competition law challenges. Academics argue that data spaces are conceived as distributed infrastructures in which interoperability, trust, and sovereignty are embedded ex ante into their technical and governance architecture.^78^ In this way, access conditions and safeguards are built into the system rather than negotiated case by case. Rather than requiring exhaustive integration of heterogeneous sources, data spaces rely on common standards, connectors, and usage policies to enable data sharing across institutional and sectoral boundaries, without ceding control and ownership by data holders. In competition-law terms, such design reduces transaction and switching costs, lowers entry barriers for SMEs, and limits the ability of powerful actors to leverage exclusive control over non-replicable datasets.^79^ By making interoperability and portability the default, data spaces curb lock-in and incumbents’ informational advantages, thus mitigating foreclosure and dominance risks through common standards, connectors, identity-and-access management, and usage policies that enable cross-sector data use without ceding control and under transparent, non-discriminatory terms.

To operationalise this architecture and prevent the re-emergence of technical fragmentation, data spaces translate these conceptual safeguards into concrete data-sharing practices by embedding the FAIR principles (Findable, Accessible, Interoperable, and Reusable) into their core design.^80^ The FAIR principles ensure that data within these federated infrastructures does not remain siloed in unstructured formats, but is actively managed through semantic interoperability and rich, machine-actionable metadata.^81^ For instance, to ensure data is Findable and Accessible, data spaces require datasets to be assigned globally unique Persistent Identifiers (PIDs) and indexed in federated catalogues, allowing new market entrants to discover available data resources without navigating proprietary, closed ecosystems.^82^

Furthermore, to guarantee Interoperability and Reusability, data spaces mandate that metadata must explicitly define usage licenses, provenance, and data formats.^83^ This robust metadata framework is essential from a competition and regulatory perspective, since it establishes traceable data flows that allow data providers to securely monitor downstream reusability. As demonstrated by recent implementations of the International Data Spaces (IDS) architecture in the healthcare sector, embedding FAIR principles within a distributed process execution environment allows for automated data usage control.^84^ In such environments, smart policies and trusted connectors verify that a data consumer is adhering to the agreed-upon contractual terms, privacy restrictions (such as GDPR), and Data Act obligations before any data processing occurs. Consequently, the integration of FAIR principles ensures that the pro-competitive promise of data spaces, cross-sectoral interoperability and market contestability, is practically grounded in technically enforceable standards that preserve data sovereignty.^85^

#### 3.3.6 Economies of scale and scope in data aggregation

The concept of Economies of Scale and Scope in Data Aggregation (ESSDA) describes the phenomenon that the larger and more diverse data pools become, the greater the value that can be extracted from their aggregation.^86^ In other words, as datasets are merged, the predictive power, efficiency, and quality of outcomes improve, creating benefits that individual, siloed datasets cannot achieve. This is particularly visible in health research, where empirical evidence from the Netherlands demonstrates that integrating health data with socioeconomic variables across multi-domain pools enhances the accuracy of health outcome predictions.^87^ The findings illustrate the pro-competitive effects of economies of scale and indicate that the social value of merged datasets is significantly higher than the value of privatised data collections. The empirical evidence indicated that integrating health data with socioeconomic data into multi-domain data pools enhances the accuracy of health outcome predictions.^88^ This process also generates network effects within the data economy, as the more data that is collected and integrated, the greater the quality and variety of services that can be offered.

Alongside scale, the non-rivalrous character of data as a product which cannot be exhausted by use, generates economies of scope that ultimately benefit consumers. The overall value to society increases, as data can be reused after collection and combined from different sources, allowing multiple parties to extract further benefits from it.^89^ However, in practice, such reuse is often impeded by classical market failures such as high transaction costs of negotiating bilateral licences, asymmetric information about the value and quality of data, and negative externalities such as privacy or security risks, which discourage voluntary sharing.^90^ By embedding interoperability ex ante, data spaces directly mitigate these failures, since common standards lower transaction costs and reduce asymmetries, while governance frameworks ensure lawful and secure data flows.^91^ “Openness” in this context refers not merely to technical availability but to the principles of non-discriminatory access and reusability, as reflected in the EU’s Open Data Directive, which requires high-value datasets to be made available in machine-readable and standardised formats.^92^ Through this architecture, economies of scope are realised under governance conditions that prevent exclusive control over data resources, ensuring that reuse enhances both consumer welfare and market contestability.

The aggregation of data from multiple domains within data spaces, whether sector specific or cross sector specific data, as part of the vision of a European single market for data, demonstrates how the ESSDA phenomenon can enhance innovation without raising competition law concerns. In conventional markets, the accumulation of vast datasets by a dominant firm may give rise to data monopolies and foreclosure risks if accompanied by an abuse of dominance in the form of exclusionary practices, such as refusal to grant access to indispensable datasets -a high bar under the essential facilities doctrine, given data multihoming's scarcity of truly unique resources-93 or discriminatory licensing, which prevent rivals from competing effectively in adjacent markets.^94^ By contrast, in data spaces the very increase in data volume strengthens pro-competitive dynamics, because of economies of scale and scope. The larger the aggregated datasets, the greater the potential for innovation, accuracy, and efficiency gains. Aggregation occurs under governance frameworks that preserve data sovereignty and ensure lawful sharing under all relevant EU regulations.^95^ In this way, more data does not equate to greater market power, but rather to more precise outcomes, enhanced innovation, and efficiency benefits that are accessible to all participants on equal terms.

#### 3.3.7 Openness and market entry

“Openness” standing at the core of European Data Spaces,^96^ is a term prima facie in favor of competition. By granting access to data that was previously siloed or monopolised, data spaces lower barriers to entry and enable smaller firms and start-ups to compete with incumbents on more equal terms. This intensifies competition in the market that leads always to increased efficiency, consumer benefit and intensifies market dynamism. 97

A second pro-competitive effect of openness is that it reduces information asymmetries between data-rich undertakings and other market actors, including consumers. Greater transparency reduces market frictions and enables effective competition in price and quality. For instance, the creation of a European Environmental Data Space would make data on energy consumption, emissions, and sustainability openly accessible to all market participants. This would empower new entrants to develop services such as personalised energy efficiency solutions or dynamic pricing models, while consumers would benefit from greater choice and the ability to make more informed decisions.^98^ As a result, new entrants could develop innovative services, such as personalized energy efficiency solutions or dynamic pricing models, while consumers would benefit from greater choice and the ability to make more informed decisions. This increased transparency enhances competition, drives better market outcomes, empowers the consumers and supports broader sustainability objectives.

Yet the legal framework shows that openness alone is not sufficient. It is closely tied to data portability rights under Article 20 GDPR^99^, which can lower switching costs and foster entry by facilitating data flows. However, portability is limited in scope: it applies only to personal data “provided” by the data subject, excludes inferred and derived data, and in practice remains underused and technically difficult to operationalise. This shows why legal rights alone are insufficient: without neutral governance through data spaces, openness risks reinforcing market concentration rather than diffusing it.^100^

Data that was once fragmented, siloed, and monopolized by dominant players will now be open and accessible for exploitation and reuse, unlocking new opportunities for competition through innovative, data-driven business models. A prime example is the European Mobility Data Space (EMDS), a key objective of EU policy. By broadening access to critical mobility datasets, the EMDS lowers entry barriers, promotes innovation, and enhances sustainability, thereby aligning openness with both competitive markets and broader public interest goals.^101^

#### 3.3.8 Data spaces, AI development and cloud integration

Moreover, European Data Spaces will play a pivotal role in enabling advanced AI techniques and supporting a dynamic and competitive marketplace for cloud and edge-based services, particularly as machine learning models increasingly depend on access to large, diverse datasets.^102^ From a competition law perspective, the pro-competitive effect lies in addressing access bottlenecks, since by making training data lawfully and interoperably available to multiple market participants, data spaces reduce the risk that only a handful of dominant undertakings can develop state-of-the-art AI models. 103 Thus, market entry barriers for SMEs and start-ups are lowered and market contestability is increased.

By enabling lawful, trusted, and interoperable data transactions across participants, data spaces provide a structured environment in which AI systems and especially Generative AI can be trained and deployed more effectively. These spaces aggregate and harmonize data from multiple sources such as public institutions, private companies, and individuals, thus addressing fragmentation and siloed data landscapes, which are among the primary barriers to robust AI development in Europe. Competition is further stimulated because such aggregation reduces duplication of costly data-collection efforts and prevents exclusive control of essential datasets, enabling a broader range of firms to innovate.^104^

Recent research illustrates how these dynamics are operationalised in practice. The *Green.Dat.AI* project^105^ has developed a Reference Architecture that integrates data spaces with AI services, including catalogue services, identity and access management, and app registries to ensure interoperability across participants. In competition law terms, such architectures mitigate the risk of exclusionary conduct by dominant firms that might otherwise rely on proprietary standards to foreclose rivals, and instead lower entry barriers by providing neutral technical conditions for access.^106^ The project has also introduced an Energy Efficiency Framework for benchmarking and optimising AI models. This framework fosters dynamic efficiency by allowing competition to take place on the merits of sustainability and performance, not simply on the computational scale of incumbents, thereby counteracting exploitative or exclusionary strategies by firms with superior infrastructure.^107^ A smart farming pilot under *Green.Dat.AI* demonstrates these effects in practice, by pooling satellite, drone, and IoT data within a common space, multiple undertakings can develop AI-based services for pest detection, irrigation, and fertilisation optimisation.^108^ Multiple undertakings can thus develop AI-based services without triggering the stringent 'indispensability' test of the essential facilities doctrine, as multihoming typically precludes data from being uniquely indispensable.^109^ This reflects the logic of the essential facilities doctrine in EU competition law, since data that could otherwise constitute a bottleneck resource is made commonly accessible under governance rules, preventing monopolisation and enabling a wider range of actors to innovate.

European Data Spaces are being closely aligned with the EU’s evolving infrastructure for cloud and edge computing.^110^ This integration is essential for supporting scalable and real-time AI applications. For competition law, the pro-competitive effect is twofold: first, interoperability between data spaces and cloud/edge infrastructures prevents vertical foreclosure by dominant cloud providers, ensuring that data access is not tied to proprietary infrastructures and second, it allows new entrants to compete on equal terms in providing time-sensitive AI solutions such as smart city systems, industrial automation, or autonomous mobility.^111^

By coupling data spaces with cloud and edge technologies, the EU aims to create a seamless data and computing continuum that can support both the training and real-time inference needs of AI systems, including the most resource-intensive Generative AI models. This contributes to dynamic efficiency in competition law terms, since lowering barriers to AI development fosters innovation-driven rivalry and prevents technological markets from tipping irreversibly in favour of a few dominant players.^112^

#### 3.3.9 Cross-sector synergies and public interest benefits

The aggregation of data across domains and sectors within European Data Spaces generates synergies that enhance both innovation and competition. Synergies include improved reuse of data, reduced redundancy between datasets, and the possibility for interoperable services across disparate sectors, by linking for example environmental, mobility and health data to produce holistic insights.^113^ From a competition law perspective, such cross-sector pooling reduces information asymmetries, mitigates duplication of data-collection costs, and enables dynamic efficiency by allowing firms to innovate in areas that no single dataset could support. By making interoperable data available across governments, municipalities, public agencies, and private undertakings, data spaces foster new forms of rivalry in markets that were previously constrained by fragmentation.

These dynamics are visible in emerging EU initiatives. For instance, by connecting destinations and hospitality providers, the European Tourism Data Space^114^ opens access to datasets that had been monopolised by global platforms, thereby lowering entry barriers and enabling smaller firms to compete in offering sustainable and competitive travel services. In a similar vein, the European Mobility Data Space^115^ pools urban mobility data, as seen in regions such as Flanders and Budapest, which allows innovators to develop applications for traffic management and emission reduction. This weakens the gatekeeping role of incumbents who previously controlled exclusive access to transport datasets, thereby enhancing market contestability. The European Health Data Space^116^, meanwhile, makes cross-border medical data and anonymised research datasets accessible, ensuring that health-tech SMEs and research institutions can participate in markets for digital health solutions without depending on dominant undertakings. Finally, the Public Procurement Data Space^117^ strengthens transparency by linking procurement data across jurisdictions, reducing information asymmetries that often entrench large contractors and opening the field to SMEs.

Taken together, these initiatives show how cross-sector data spaces translate into pro-competitive effects: they dismantle entry barriers by diffusing access to essential datasets, counteract risks of foreclosure where incumbents once controlled data as bottleneck resources, and stimulate dynamic efficiency by allowing innovation across sectors such as tourism, mobility, health, and public procurement. In doing so, they demonstrate that data spaces not only deliver public interest outcomes but also directly reinforce competition law objectives by preventing market tipping and fostering sustainable rivalry**.**

#### 3.3.10 Impact outlook

When considering the cumulative benefits, it becomes clear that European Data Spaces are instrumental in strengthening Europe’s digital resilience and competitive landscape. By reducing dependency on foreign data infrastructures and external technology platforms, they pave the way for the creation of secure, sovereign, and interoperable data ecosystems within the EU. This sovereignty empowers the Union to safeguard its core values, protect critical infrastructure, and assert control over its economic and digital future. Relying on the capabilities, resources, and innovations of the European internal market enhances operational efficiency across Member States and ensures that the economic value generated from data remains within the Union. This strategic autonomy allows the EU to shape its own digital trajectory, while also fostering fair competition, innovation, and trust. As a result, citizens benefit directly; they gain access to goods and services developed within the Union, aligned with European standards and regulations, and priced in accordance with the internal market’s own dynamics of supply and demand. In this way, European Data Spaces not only support technological advancement but also serve as a catalyst for inclusive economic growth, consumer empowerment, and long-term digital sovereignty.

In competition law terms, such autonomy prevents dependency on gatekeeping platforms outside the EU and thereby reduces the risk of structural foreclosure in data-driven markets. By leveraging the capabilities and resources of the internal market, Member States benefit from increased efficiency, while firms of all sizes gain a level playing field in accessing and exploiting data.

A central element of this impact is trust and legal certainty. European data regulation currently spans the GDPR, the Data Governance Act and the Data Act, which sometimes impose conflicting requirements: the GDPR stresses data minimisation and purpose limitation, while the DGA promotes free reuse and circulation of data across the Union. Data spaces are intented to function as a structural solution to this tension, embedding interoperability, compliance, and privacy-preserving techniques into their design, even though the effectiveness of these techniques in large-scale deployments remains to be empirically validated.^118^

In early data space deployments, this promise is operationalised not by unrestricted data circulation, but by combining architectural constraints with concrete privacy-preserving techniques. Sectoral initiatives such as health and energy data spaces experiment with federated analytics, where models are trained locally and only model updates, rather than raw personal data, are exchanged across nodes, thereby limiting the need for cross-border data transfers while still enabling joint insights.^119^ At the same time, a growing body of work on trusted data spaces proposes the use of cryptography-based methods, including secure multi-party computation, homomorphic encryption and private set intersection, to support privacy-preserving queries and computations on distributed datasets, so that parties can learn global statistics without exposing underlying records.^120^ In practice, these techniques are complemented by more classical measures already visible in data-space pilots, such as encryption in transit and at rest, strong authentication and authorisation at connector level, and the confinement of processing within certified secure environments.^121^ Yet, as literature concedes, such approaches still face performance bottlenecks, implementation complexity and residual re-identification risks, which means that privacy-preserving data spaces should be understood as an evolving instantiation of “data protection by design” under the GDPR rather than as a fully solved tension between extensive data sharing and fundamental rights.^122^

Thus, data spaces overall reduce regulatory uncertainty and lower the transaction costs of negotiating lawful access. For competition law, the fact that firms and especially SMEs and start-ups, can participate in cross-border data markets without incurring prohibitive legal risks or relying on costly bilateral contracts with dominant incumbents, this is critical. In short, data spaces transform regulatory fragmentation into a governance framework that supports predictability, contestability, and fair rivalry.

The benefits are therefore systemic and go beyond the correction of isolated market failures usually addressed by competition law enforcement. By democratising access to data, European Data Spaces ensure that not only large enterprises but also SMEs, start-ups, consumers, and public bodies can benefit from innovation. This inclusivity strengthens competition on the merits by diffusing innovation incentives broadly rather than concentrating them in a few dominant players. Therefore, on the one hand competition law interventions focus on correcting specific market failures, and on the other, data spaces operate as a structural, pro-competitive policy tool by delivering broad-based benefits such as sustainability, digital transformation, and social inclusion, while simultaneously embedding the governance and trust necessary for markets to remain open, contestable, and dynamic.

## 4 Characteristics of Pro-Competitive Data Spaces

The pro-competitive benefits of data spaces, as outlined above, are made possible by a set of core characteristics that enable them to promote efficiency and enhance both consumer and societal welfare. These benefits do not arise automatically but depend on the deliberate design of data spaces to support openness, interoperability, trust, and fair access. These characteristics fall under: technical infrastructure, interoperability, neutrality and trust and economic dynamics.

### 4.1 Technical infrastructure

#### 4.1.1 Interoperability by design

To begin with, European Data Spaces are designed on the basis of common technical standards and open interface principles that facilitate interoperability across systems and sectors.^123^ This orientation is evident in the proposed European Health Data Space (EHDS)^124^, which introduces binding requirements for Member States to adopt common formats, terminologies, and standards for electronic health records. The EHDS aims to create a unified framework where patients, healthcare providers, and innovators can access and reuse data across borders in a seamless and secure way. By standardising data structures and mandating interoperability services, the EHDS prevents health data from being locked into fragmented national systems or proprietary platforms,^125^ thereby limiting the ability of dominant electronic health record providers to engage in exclusionary practices such as technical lock-in or refusal to grant access to indispensable patient data. In competition law terms, this reduces the risk of “essential facility” situations in digital health markets, where control over unique datasets could otherwise allow incumbents to foreclose rival health-tech firms and restrict innovation in diagnostics, telemedicine, or AI-driven treatments.^126^

By embedding interoperability, data spaces prevent the exclusionary tactics of past data and tech monopolies and take an opposite approach. The importance of interoperability is also highlighted by past antitrust enforcement. In the landmark Microsoft case,^127^ for instance, Microsoft was fined for refusing to share critical interoperability information with rivals that was considered to be an abuse reinforcing Microsoft’s dominance. On the contrary of this business practice, data spaces operate through APIs, shared data and compatibility from the start,^128^ which is a business model that doesn’t allow providers to withhold information and protocols with the potential to drive competitors out of the market. This design fosters a level playing field in the data market and ensures the integration and exchange of data on equal terms.

#### 4.1.2 Federated cloud and data sovereignty

Digital sovereignty becomes increasingly critical as Europe seeks to reduce dependence on foreign digital infrastructure and maintain control over strategic data assets. By 2030, enterprise cloud data flows in European countries are expected to grow 2-3 times, underscoring the importance of sovereign cloud infrastructure for business growth.^129^ The federated approach enables European organizations to maintain data locality requirements while participating in cross-border data sharing initiatives.^130^

In addition, a core pro-competitive technical design is the federated cloud model that fosters data sovereignty, as has been exemplified by initiatives like GAIA-X. Instead of concentrating data in one proprietary platform, data spaces link many clouds and data providers in a decentralized network.^131^ Decentralization involves shifting power, authority, or decision-making from a single central body to multiple actors, allowing control to be shared across various participants rather than concentrated in one entity. Data stays with its owner until needed, and only the necessary data is shared or queried when a use-case arises. This architecture ensures data sovereignty given that each provider retains control over who can access their data and under what conditions, enforced through usage rights and access.^132^ The result is a trustworthy environment where participants feel safe sharing data without ceding ownership. Technically, common security standards, identity management, and usage policy enforcement are built in to uphold these sovereignty and trust guarantees. The Bundeskartellamt, in its 2022 decision on Catena-X, explicitly recognised that such decentralised infrastructures could strengthen competition in cloud services, provided that standards are developed transparently and on FRAND terms.^133^

#### 4.1.3 Open standards and portability

Data spaces provide standardized specifications for data exchange that are vendor-neutral and technology-agnostic.^134^ Three key components of data sharing are established, which if met, ensure that participants can collaborate and exchange information seamlessly, without being dependent on any particular vendor's proprietary systems or formats: (a) “catalogs” which use metadata standards, to describe what data is available (b) common language for expressing how data can be used, including any restrictions or permissions and (c) automated negotiation of data usage agreements between parties.^135^

By using open, standard protocols and formats, data spaces actively prevent vendor lock-in and foreclosure effects. More specifically, all members can port their data or switch service providers without undue friction, that is a deliberate pro-competitive measure, since users cannot be trapped in a closed ecosystem by a dominant player. Open participation and data portability mean a newcomer can join a data space and interconnect without needing permission from an incumbent. This disrupts the lock-in advantage that big tech platforms often enjoy. In practice, data spaces require data to be made available in standardized, machine-readable formats and often adopt open-source reference architectures, so that all tools and services remain compatible.^136^ By removing proprietary barriers, data spaces ensure that no single firm can exploit control over data formats to exclude rivals. Notably, the EU’s case against Google Shopping highlighted how a dominant platform’s self-preferencing and closed ecosystem harmed competition.^137^ In contrast, a well-designed data space would mitigate such risks by guaranteeing equal access and neutral display of data from all providers without the risk of a dominant player engaging in self-preferencing activities.

This design choice aligns with the European Commission’s 2021 comfort letter to Gaia-X, where DG Competition emphasised that open and non-discriminatory participation in standard-setting reduces antitrust risk and ensures fair access for all stakeholders.^138^ Open interfaces and machine-readable standards not only reduce switching costs but also steer data uses towards pro-competitive outcomes, and by making non-discriminatory access the norm, data spaces prevent the escalation of surplus-extraction strategies while making firms compete on quality, not on opacity.^139^

### 4.2 Governance model: Trust, neutrality and fair access

#### 4.2.1 Sovereignty by design

The second most prominent characteristic of data spaces that fosters efficient competition, is their governance model, which is designed to generate trust and ensure fair participation. Pro-competitive governance in data spaces is centred around empowering data providers with control and confidence over how their data is shared and reused. It has been argued that the legal architecture of data spaces deliberately “puts the legal instruments and tools in the hands of each participant,” combining freedom of contract with compliance (antitrust, GDPR, cybersecurity) and supporting this with usage-control technology that lowers transaction costs and strengthens enforcement.^140^ These mechanisms make clear under which purposes and terms data can be accessed, while also requiring providers to respect third-party rights such as copyright and personal data protection. Scholars further note that usage control can travel with the dataset (authorised users, permitted purposes, duration, onward sharing), complementing contracts rather than replacing them.^141^

In practice, many data spaces rely on data transaction agreements or contractual templates that embody the principle of data sovereignty, allowing providers to set conditions for use.^142^ These mechanisms make clear under which purposes and terms data can be accessed, while also requiring providers to respect third-party rights such as copyright and personal data protection.^143^ By doing so, providers retain control as “original owners” of their data without losing sight of external legal obligations. This sovereignty-by-design reduces the fear of losing control, making participants more willing to share data and thereby enlarging the ecosystem. From a competition law perspective, this governance approach prevents any one party from unilaterally exploiting shared data in an exclusionary way, since usage is channeled through collectively agreed technical and contractual safeguards.^144^

#### 4.2.2 Neutral intermediaries and fair management

Trusted intermediaries and neutral conveners that act independently, operate the data space’s infrastructure in a neutral manner and enhance the decentralized and fair management of the data space. They are often referred to as “data intermediaries” and have the responsibility to remain neutral by separating their data intermediation services from any other commercial and monetary use of data that goes beyond their role as facilitators.^145^ The intent is to organize data spaces in an open and collaborative way, without any intermediary accumulating significant market power. In essence, the governance model mandates that the data space operator or convenor is a neutral party enforcing the rules, rather than a dominant firm exploiting the data. This builds trust among participants that the platform itself will not favor one contributor over another. Compliance with legal frameworks, from privacy (GDPR) to competition law, is built into the governance. For example, the Data Governance Act increases trust in data sharing by mandating neutrality and imposing fiduciary duties on intermediaries and requiring adherence to EU laws when handling data,^146^ which are principles that map directly onto data-space operators. Overall, such governance structures ensure fair, decentralized management of the data space, where rules apply equally to all and no participant (including the operator) can secretly tilt the playing field. The creation of Cofinity-X, backed by BMW, Mercedes-Benz, Volkswagen, BASF, SAP and others, is a practical illustration of the above.^147^ It operates Catena-X as a neutral gateway without storing data itself, offering instead a marketplace and access framework where each participant retains control.

#### 4.2.3 Transparency and accountability

With regard to transparency and accountability as a prime characteristics of data spaces, it is vital that all participants (data users, holders, providers, subjects) know “the rules of the game”. The criteria for participation, pricing, data quality standards, and governance policies should be openly published, which makes competition in the data market predictable and fair. The principle of transparency is foundational in all EU initiatives^148^ that data spaces aim to simplify, such as the EU’s Open Data Directive^149^ that explicitly builds on transparency and fair competition in the reuse of public sector information. It requires public bodies to make key datasets available in machine-readable formats and to avoid exclusive agreements that lock up data. In fact, public-sector data made available to private firms must be under non-discriminatory conditions, with no exclusive access. The Commission’s Data Strategy frames data spaces precisely as governance frameworks that “overcome legal and technical barriers to data sharing” by combining tools, common rules and interoperability—so transparency and predictability are design features, not afterthoughts.^150^ Data spaces mirror these values by publishing their governance frameworks and participation criteria publicly,^151^ ensuring that all data providers and users are treated under the same terms. This transparency prevents dominant players from engaging in covert self-preferencing or exclusion. Clear audit trails, compliance checks, and accountability mechanisms (sometimes via independent oversight boards or the upcoming European Data Innovation Board under the Data Governance Act)^152^ further ensure that no participant can secretly break the rules without detection. Participants collectively benefit from an environment where trust and fair play are enforced, encouraging more open data sharing which ultimately fuels healthy competition.

#### 4.2.4 Fair Access and non-discrimination

Lastly, in the category of governance and trust, a fundamental pro-competitive characteristic of the design of data spaces is fair access and non-discrimination. The requirements to access are transparent as analysed above, and fair access refers to the ability of any entity that meets these publicly available criteria (such as common legal terms and technical standards) can join the data space without facing arbitrary barriers. Since all terms of access and pricing are the same for both smaller entities and multinational entities, all participants are subject to non-discriminatory conditions which is in line with EU competition law precedents. This principle was underscored in DG Competition’s guidance to Gaia-X, which stressed that safeguards such as equal voting rights in working groups, transparent governance, and published rules of participation are central to ensuring adequate access and avoiding covert exclusion.^153^

In cases like *Magill*^154^ and *IMS Health*^155^, the EU enforced that certain indispensable data (TV listings in Magill, and a pharmaceutical sales data format in IMS) had to be licensed to would-be competitors on non-discriminatory terms because outright refusal would eliminate competition. Data spaces do not mandate such compulsory access. Instead, participation and data sharing are voluntary, but once entities choose to join, they operate under common governance rules that ensure resources made available within the space are shared on transparent and non-discriminatory terms. In this way, data spaces embed safeguards that mirror the objectives of competition law: they make it infeasible for a participant to corner a dataset or to favour some partners over others once data is brought into the system. Every participant, regardless of size, has a fair shot at leveraging shared data to create value, which encourages competition on the merits (innovation, quality of service) rather than control of data bottlenecks. This voluntary yet rule-based structure is precisely why data spaces are often described as “soft-market interventions”, as they shape access conditions and reduce competitive risks without imposing compulsory licensing or coercive remedies.

### 4.3 Economic dynamics

#### 4.3.1 Lowering transaction costs and solving coordination problems

Building on the analysis of the pro-competitive characteristics of data spaces, it is important to highlight their potential to address the market failures that have historically hindered data sharing. In ordinary markets, sharing data across organizations involves high transaction costs due to the efforts of finding partners, negotiating contracts for each data exchange, ensuring technical compatibility, etc., that can be prohibitively costly. Data spaces act as market intermediaries that drastically lower these transaction costs.^156^ By providing a ready-made infrastructure (standardized interfaces, legal templates, trust frameworks), a data space makes it much easier and cheaper for any two parties to exchange data on a one-to-one or many-to-many basis. This reduction in friction enables more firms to participate in data-driven innovation, including smaller companies that otherwise couldn’t afford complex bilateral data deals with high transaction costs. In effect, data spaces solve coordination problems. They pool disparate datasets or connect data providers with users under a common institutional setup, thereby addressing the *“excessive exclusivity”* problem where valuable data sits siloed due to fear of misuse or high sharing costs.^157^ Data spaces capture these benefits by enabling data pooling and reuse that wouldn’t happen in a high-transaction-cost environment. This boosts competition by allowing new products and services to emerge from combined data sources. In short, data spaces turn inaccessible or isolated data into a shared resource, levelling the playing field and spurring innovation across an entire sector.

#### 4.3.2 From competition for data to competition on data

Moreover, the vision of a European Data Space goes beyond mere bilateral exchanges that are restricting. Common data pools that are accessible to all members with agreed terms, creates a valuable resource. Taking the example of the European Health Data Space, if multiple hospitals join their data together in a pool, then health data could power AI diagnostics that no entity could have achieved alone. By enabling such collaboration, data spaces help overcome the problem where no single firm has enough data (or incentive) to invest in certain innovations. Economic theory supports this pooling effect: without safeguards, data-driven markets tend to “tip” towards monopolisation because incumbents benefit from self-reinforcing data advantages, but if data is shared, the tipping mechanism is neutralised and competition shifts to how effectively firms can exploit commonly available data rather than how much data they exclusively control.^158^ Therefore, the economic dynamic shifts from competition for data (exclusive hoarding) to competition on data (how effectively data is used).^159^

All participants can draw insights from the larger pooled datasets, and then they compete in how effectively they use those insights in their products. This reduces entry barriers for innovators, since even a startup in a data space might access industry-wide datasets that only incumbents historically possessed, thereby giving the startup a chance to compete in the offering of a good or service. The data space thus addresses information asymmetries and concentration of data that often lead to winner-take-all effects which hinders competition. By democratizing access to data, more firms can compete on service quality, analytical capability, and customer understanding. This corresponds to what is described in academic literature as eliminating the “domino effect” of market tipping, where dominance in one data-driven market can be leveraged into another and therefore collective sharing undercuts this path to monopoly and sustains multi-firm rivalry.^160^ Importantly, many data spaces operate under rules that prevent over-centralization of the pooled data’s benefits. For example, governance might prohibit any single participant from unilaterally commercializing the pooled dataset without returning value to contributors, or might rotate leadership roles, ensuring collaborative benefits rather than domination by the largest member. Overall, the pooling dynamic, if managed well, can simulate a more perfect market where even smaller players can innovate using big data, driving competitive pressure on larger firms.

#### 4.3.3 Voluntary sharing and decentralisation as a structural counterweight

Data spaces also have the potential to mitigate monopolistic behaviour and foreclosure in data markets, though not by imposing legal obligations to share data. Unlike instruments such as the Digital Markets Act (DMA), data spaces are voluntary infrastructures. Dominant firms cannot be forced to contribute data, but they are provided with structured, trusted environments that create incentives to do so. For instance, shared governance, contractual templates, and neutral intermediaries lower transaction costs and build trust, encouraging even powerful actors to participate when this enables access to complementary datasets or compliance benefits. In this sense, data spaces reduce the likelihood of monopolisation indirectly, by making voluntary sharing more attractive and by ensuring that, once inside, all participants are subject to transparent and non-discriminatory conditions.

From a competition law perspective, this means data spaces do not prohibit dominance in the abstract, but they constrain the ability of a participant, whether large or small, to use shared data in an exclusionary manner. The safeguards resemble FRAND-like obligations and while firms remain free to retain their proprietary datasets, any data they bring into the space must be made available on equal terms. This creates a structural balance that preserves contestability without requiring compulsory licensing.

The Meta case illustrates the risks that arise in the absence of such governance. More specifically, the Bundeskartellamt found that Meta abused its dominance by combining Facebook, Instagram, and WhatsApp data to strengthen market power. A data space could not prevent Meta from pooling its own proprietary datasets, but it shows why alternative infrastructures are needed. By offering a neutral, federated environment for data sharing, data spaces reduce dependence on unilateral data ecosystems controlled by a single gatekeeper. In contrast, the Bundeskartellamt’s approval of Catena-X demonstrates how federated infrastructures can be designed to support competition, while CADE’s prohibition in Brazil highlights the need for strong safeguards against covert collusion or information exchange among incumbents.

The competitive effect of data depends on how it influences firm behaviour. Data is unilaterally pro-competitive (UPC) when it raises the value or quality of offers, such as product improvement or innovation, and unilaterally anti-competitive (UAC) when it mainly enables surplus extraction, like price discrimination. European data spaces steer data uses toward the UPC regime through interoperability, transparency, and privacy-preserving governance, ensuring that data aggregation fuels innovation and consumer benefit rather than market dominance.^161^

Data spaces therefore counter monopolisation not through compulsion but through decentralization. They distribute control among many actors, ensure data remains at source, and make participation conditional on neutrality and interoperability. In a federated data space, there is no central hoarding of all data by one company, since each data provider keeps control and can even participate in multiple data spaces.^162^ This pluralism means the market is not dependent on a single gatekeeper and no data monopolies emerge within the space itself. By allowing multiple cloud providers and service nodes, users can choose or switch between them, injecting competitive pressure on providers to offer better terms.

## 5 Conclusion

Safeguarding competition law is not incidental but integral to the design of European data spaces. By embedding openness, interoperability, neutrality, and non-discrimination into their technical and governance architecture, data spaces respect competition law principles by design and advance the objectives of market contestability, consumer welfare, and innovation. Their federated infrastructure prevents lock-in and foreclosure, their governance frameworks empower participants to share data without ceding sovereignty, and their economic dynamics shift rivalry from the exclusive hoarding of data to its effective and innovative use.

In this sense, data spaces embody a dual contribution. They provide structural solutions to market failures ex ante, while also shaping a governance environment in which competition law can operate more effectively ex post. Their architecture transforms data from a potential source of dominance into a shared resource for innovation, supporting the vision of data as a “fifth freedom” within the EU internal market. If designed and governed with vigilance, European data spaces will not only strengthen digital sovereignty but also ensure that competition in data-driven markets remains open, fair, and sustainable creating the foundation for long term efficiency.

Ultimately, the long-term success of data spaces turns on keeping these safeguards operative in practice. The Bundeskartellamt’s 2022 approval of Catena-X shows how decentralised standards and federated governance can strengthen competition across cloud and automotive supply chains when participation is transparent and on FRAND terms.^163^ The Brazilian CADE decision in 2023, by contrast, underscores what fails without robust governance.^164^ Taken together, these cases do not counsel retreat but rather they map the guardrails. With clear participation rules, neutral intermediation, and compliance guidelines, data spaces can scale safely and realise their core promise of shifting rivalry from data hoarding to data-driven innovation.Based on this analysis, this paper offers three explicit recommendations for policymakers and industry stakeholders engaged in the deployment of European data spaces to ensure these guardrails hold: First, governance must remain strictly decoupled from data hosting. To prevent the re-emergence of proprietary ecosystems, the entities acting as federators or trust anchors within a data space must function as genuinely neutral intermediaries. They must be legally and technically prohibited from leveraging their infrastructural position to access, monetise, or gatekeep the underlying data, ensuring they do not devolve into dominant platforms under Article 102 TFEU.

Second, antitrust authorities must issue tailored guidance on data spaces. While the Commission’s *Horizontal Guidelines* address data pools generally, the unique, real-time, federated nature of data spaces requires specific safe harbours. Guidelines should explicitly clarify how collusion and standard-setting foreclosure can be avoided through specific algorithmic and architectural designs (such as mandatory anonymisation routing or time-lagging of sensitive pricing data), thereby providing the legal certainty necessary for market participants to collaborate.

Third, participation incentives for data-rich incumbents must be carefully engineered. As literature correctly notes, voluntary data spaces risk underutilisation if dominant players who already possess massive proprietary datasets refuse to participate.^165^ To overcome this, policymakers and data space architects must align participation with concrete regulatory and commercial incentives. First, data spaces must be positioned as efficiency mechanisms for regulatory compliance. For example, under the Corporate Sustainability Reporting Directive (CSRD) and the Corporate Sustainability Due Diligence Directive (CSDDD), large enterprises are legally obligated to trace and report environmental and social impacts across their entire upstream and downstream supply chains.^166^ Extracting this granular data through bilateral contracts is prohibitively expensive. Data spaces like Catena-X incentivise incumbents to join by providing a standardised, automated infrastructure for exchanging Product Carbon Footprint (PCF) data, drastically lowering compliance costs. Second, participation must offer operational resilience. The disruptions of recent years have proven that even dominant manufacturers lack visibility beyond their Tier-1 suppliers. Data spaces incentivize participation by offering multi-tier traceability, allowing incumbents to predict and mitigate supply chain shocks. Finally, regarding AI development, even data-rich incumbents suffer from domain-specific data silos. Joining cross-sectoral data spaces provides these actors with access to the high-quality, heterogeneous data pools required to train next-generation AI models, proving that the commercial value of cross-sectoral synergies outweighs the perceived risks of abandoning proprietary data hoarding.

The European Union has set a clear target that aligns with the role of data spaces in scaling trusted sharing: by 2030, the EU’s share of the data economy should at least correspond to its economic weight, achieved by choice through an attractive European data environment.^167^ The future-oriented design of European data spaces represents more than a technical infrastructure project, since it embodies Europe's commitment to human-centric digital transformation that preserves fundamental rights while unlocking the economic and societal benefits of data-driven innovation. As these systems mature and interconnect, they will form the digital backbone supporting Europe's transition to a sustainable, competitive, and sovereign digital economy.

## Ethics Statement

The author confirms that she has read and complies with the ethical requirements for publication in this Journal. Furthermore, the current work does not involve human subjects, or any data collected from social media platforms.

## Footnotes

1 Pascal D König, ‘Fortress Europe 4.0? An analysis of EU data governance through the lens of the resource regime concept’ (2022) 8(4) *European Policy Analysis* 484, 489–490.

2 European Commission, *A European Strategy for Data* COM (2020) 66 final, 19 February 2020, https://eur-lex.europa.eu/legal-content/EN/TXT/?uri=CELEX%3A52020DC0066.

3 Lars Nagel and Douwe Lycklama, ‘Design Principles for Data Spaces’ (Zenodo 2021) 76–79 https://zenodo.org/record/5244997.

4 European Commission, *Common European Data Spaces* (Shaping Europe’s Digital Future, 9 July 2025) https://digital-strategy.ec.europa.eu/en/policies/data-spaces.

5 Boris Otto, Michael ten Hompel and Stefan Wrobel (eds), Designing Data Spaces: The Ecosystem Approach to Competitive Advantage (Springer International Publishing 2022) 52-54 https://link.springer.com/10.1007/978-3-030-93975-5.

6 *ibid* 8.

7 European Commission, *A European Strategy for Data,* n2, 5.

8 European Commission, *A European Strategy for Data,* n2.

9 *ibid*.

10 European Parliament and Council, *Data Governance Act* (Regulation (EU) 2022/868) (OJ L 152, 3 June 2022) recital 2.

11 DSSC, *Glossary: 1.3 Definition of data space and definition of synergy* (24 October 2024) https://dssc.eu/space/DSSE/791740421/1.3+Definition+of+data+space+and+definition+of+synergy.

12 König, n1.

13 DSSC, *Governance Building Blocks* (7 March 2025) https://dssc.eu/space/BVE2/1071253634/Governance+Building+Blocks.

14 European Union, *When Open Data Meets Data Spaces* (data.europa.eu, 2022) https://data.europa.eu/en/publications/datastories/when-open-data-meets-data-spaces.

15 International Data Spaces Association, *International Data Spaces: Governance and Architecture* (IDSA, 2021) https://www.internationaldataspaces.org/.

16 Nagel and Lycklama, ‘Design Principles for Data Spaces’ n 3, 76.

17 Hubert Tardieu, ‘Role of Gaia-X in the European Data Space Ecosystem’ in B Otto et al (eds), *Designing Data Spaces* (Springer 2022) ch 4, 52-54 https://link.springer.com/content/pdf/10.1007/978-3-030-93975-5_4.pdf.

18 Manlio Bacco and others, ‘What Are Data Spaces? Systematic Survey and Future Outlook’ (2024) 57 Data in Brief 110969 <https://linkinghub.elsevier.com/retrieve/pii/S2352340924009314>.

19 DSSC Glossary,n11.

20 Centre of Excellence for Data Sharing & Cloud, ‘SCSN and Catena-X: How to facilitate interoperability between data spaces’ (https://coe-dsc.nl/scsn-and-catena-x-how-to-facilitate-interoperability-between-data-spaces/

21 SCSN (Smart Connected Supplier Network), *SCSN – Smart Connected Supplier Network* (May 2024) https://manufacturingdataspace-csa.eu/wp-content/uploads/2024/05/SCSN-Smart-Connected-Supplier-Network.pdf

22 B Steiner and C Münch, ‘Leveraging Digital Data Spaces in Purchasing and Supply Management: Paving the Way to the Circular Economy Exemplified by Catena-X’ (2024) *Journal of Purchasing and Supply Management* 30(3), 100933, 5 https://doi.org/10.1016/j.pursup.2024.100933.

23 Centre of Excellence for Data Sharing & Cloud, n20.

24 DSSC, Data Space Design Principles: Version 2.0 (September 2024) https://dssc.eu/download/attachments/766181377/Data%20Space%20Design%20Principles.pdf.

25 European Commission, *Common European Data Spaces*, n4.

26 *ibid*.

27 Bacco and others, n18.

28 Consolidated Version of the Treaty on the Functioning of the European Union [2016] OJ C202/47, arts 26, 28–66.

29 Bart Custers and Gianclaudio Malgieri, ‘Priceless Data: Why the EU Fundamental Right to Data Protection Makes Data Ownership Unsustainable’ (2022) 45 *Computer Law & Security Review* 4 https://doi.org/10.1016/j.clsr.2022.105683.

30 European Commission, *A European Strategy for Data* n2.

31 *ibid*.

32 European Commission, ‘Data Governance Act explained’ (Shaping Europe’s Digital Future, 11 October 2024) <https://digital-strategy.ec.europa.eu/en/policies/data-governance-act-explained>.

33 E Murati, ‘Overlooking digital collusion risks in the EU’s agenda for a single European data space(s)’ (2025) 21(1) *European Competition Journal*.

34 Nicolo Zingales, ‘Data Collaboratives, Competition Law and the Governance of EU Data Spaces’ (31 July 2021) forthcoming in I Kokkoris (ed), *Research Handbook in Competition Enforcement* (Edward Elgar) https://ssrn.com/abstract=3897051.

35 Communication from the Commission, *Guidelines on the applicability of Article 101 of the Treaty on the Functioning of the European Union to horizontal cooperation agreements* OJ C 259/1, paras 377–378.

36 *ibid*, paras 384–385; L Zoboli and M Bernatt, ‘The Interplay of Data Protection, Competition Law, IP Law and Data Sharing’ in L Zoboli and M Bernatt (eds), *Data Sharing Regulation in Europe* (Routledge 2025) 185.

37 Murati, n33, 65–66.

38 *ibid* 66–67.

39 *Horizontal Guidelines*, n35, para 379 (noting that algorithms can become a "device to facilitate collusion" by increasing market transparency and making punishment mechanisms more effective).

40 M K Borowicz, ‘Competition, standards, and EU data spaces’ (2025) 62 *Data in Brief* 111964, 4.

41 *ibid* 8 (highlighting how incomplete API specifications under PSD2 allowed banks to engage in exclusionary data gatekeeping).

42 Case AT.40462 *Amazon Marketplace* C(2022) 9442 final; Case C-252/21 *Meta Platforms and Others v Bundeskartellamt* EU:C:2023:537; Zoboli and Bernatt, n36, 186.

43 *Ibid*.

44 *Horizontal Guidelines*, n35, para 382 (noting foreclosure risks when data is of strategic importance and covers a significant market share).

45 See Case C-7/97 *Oscar Bronner GmbH & Co. KG v Mediaprint* ECR I-7791; Zoboli and Bernatt, n36, 190.

46 *Horizontal Guidelines,* n35, paras 408, 418.

47 Zingales, n34; Borowicz, n40, 6.

48 DSSC, Governance Building Blocks, n13.

49 *ibid*.

50 Data Space Support Centre, *Introduction — Key Concepts of Data Spaces* (DSSC) https://dssc.eu/space/BVE2/1071251613/Introduction%2B-%2BKey%2BConcepts%2Bof%2BData%2BSpaces.

51 Tardieu, n17, ch 4.

52 F Schöppenthau, F Patzer, B Schnebel, K Watson, N Baryschnikov, B Obst, Y Chauhan, D Kaever, T Usländer and P Kulkarni, ‘Building a Digital Manufacturing as a Service Ecosystem for Catena-X’ (2023) 23 *Sensors* 7396, 3–4 https://doi.org/10.3390/s23177396.

53 *ibid*.

54 Marko Jurmu et al, ‘Exploring the Role of Federated Data Spaces in Implementing Twin Transition within Manufacturing Ecosystems’ (2023) 23 *Sensors* 4315 https://www.mdpi.com/1424-8220/23/9/4315.

55 Inge Graef and Jens Prüfer, ‘Governance of Data Sharing: A Law & Economics Proposal’ (2021) 50 *Research Policy* 104330, 8–10.

56 Zingales, n34.

57 *ibid*.

58 *ibid*.

59 Felix Beyvers and others, ‘Towards FAIR and Federated Data Ecosystems for Interdisciplinary Research’ (2025) https://arxiv.org/abs/2504.20298.

60 Björn Lundqvist, ‘An Access and Transfer Right to Data—From a Competition Law Perspective’ (2023) 11 *Journal of Antitrust Enforcement* i57, i66–i67.

61 European Commission, *European Health Data Space Regulation (EHDS)*https://health.ec.europa.eu/ehealth-digital-health-and-care/european-health-data-space-regulation-ehds_en.

62 European Commission, *European Mobility Data Space* (2022), https://ec.europa.eu/info/law/better-regulation/have-your-say/initiatives/13566-Transport-data-creating-a-common-European-mobility-data-space-communication-_en.

63 European Commission, *Blueprint proposal for the Common European Agricultural Data Space* (Shaping Europe’s digital future, 2 May 2024) https://digital-strategy.ec.europa.eu/en/library/blueprint-proposal-common-european-agricultural-data-space.

64 Björn Lundqvist, ‘Data Access and Portability and EU Competition Law’ (CPI Antitrust Chronicle November 2020) 3–4.

65 Jacques Crémer, Yves-Alexandre de Montjoye and Heike Schweitzer, *Competition Policy for the Digital Era: Final Report* (European Commission, Publications Office of the European Union 2019) 44–55, doi:10.2763/407537.

66 Carlo Zappa and others, Connecting Data Spaces and Data Marketplaces: A Comprehensive Approach to Build Trust in Data Sharing (2022) 134–135.

67 *AWS Data Exchange* (Amazon Web Services) https://aws.amazon.com/data-exchange/; *Azure Marketplace* (Microsoft) https://azuremarketplace.microsoft.com; *Google Cloud Public Datasets* (Google Cloud) https://cloud.google.com/datasets.

68 Zappa and others, n43, §2.1.

69 Consolidated Version of the Treaty on the Functioning of the European Union [2016] OJ C202/88, art 102.

70 DSSC, Governance Building Blocks,n13.

71 European Commission, *European Mobility Data Space* (2022), https://ec.europa.eu/info/law/better-regulation/have-your-say/initiatives/13566-Transport-data-creating-a-common-European-mobility-data-space-communication-_en.

72 European Commission, *European Health Data Space Regulation (EHDS)*https://health.ec.europa.eu/ehealth-digital-health-and-care/european-health-data-space-regulation-ehds_en.

73 Statista, ‘Volume of Data Created, Captured, Copied, and Consumed Worldwide from 2010 to 2023, with Forecasts from 2024 to 2028 https://www.statista.com/statistics/871513/worldwide-data-created.

74 National Institute of Standards and Technology (NIST), *NIST Big Data Interoperability Framework: Volume 1, Definitions* (Special Publication 1500-1r2, 2019) 11, 16, 18, 23, 31–32 https://doi.org/10.6028/NIST.SP.1500-1r2.

75 M Serrano and others, ‘Data Space Best Practices for Data Interoperability in FinTechs’ in *Connecting Data Spaces and Data Marketplaces: A Comprehensive Approach to Build Trust in Data Sharing* (2022) 252–253.

76 OECD, *Going Digital to Advance Data Governance for Growth and Well-being* (OECD Publishing 2022) 8–10, 30, 32–34 https://doi.org/10.1787/e3d783b0-en.

77 Bertin Martens and others, Business-to-Business Data Sharing: An Economic and Legal Analysis (JRC Digital Economy Working Paper 2020-05, European Commission 2020) 25–27.

78 S Gieß, T Schoormann and F Möller, ‘A Classification of Design Options for Data Spaces’ in M Heisel, L Taveter and A Felfernig (eds), Perspectives in Business Informatics Research (Springer 2025) 46–61.

79 Wolfgang Kerber and Heike Schweitzer, ‘Interoperability in the Digital Economy’ (2017) 8(1) JIPITEC 39, 52 https://www.jipitec.eu/jipitec/article/download/190/185/698.

80 M Wilkinson and others, ‘The FAIR Guiding Principles for Scientific Data Management and Stewardship’ (2016) 3 Scientific Data 160018 https://doi.org/10.1038/sdata.2016.18.

81 Nagel and Lycklama, ‘Design Principles for Data Spaces’, n3, 65-77.

82 M Hauff and others, ‘FAIRness in Dataspaces: The Role of Semantics for Data Management’ (2024) CEUR Workshop Proceedings, The Second International Workshop on Semantics in Dataspaces, 26–27 May 2024, Hersonissos, Greece.

83 S Steinbuss and others, *Semantic Interoperability in Data Spaces* (International Data Spaces Association 2025) https://doi.org/10.5281/zenodo.17630664.

84 M Klötgen and others, ‘A Trusted Data Sharing Environment based on FAIR Principles and Distributed Process Execution’ in *Proceedings of the 15th International Joint Conference on Biomedical Engineering Systems and Technologies - HEALTHINF* (SciTePress 2022) 583, 584–586.

85 Otto and others, n5, 238–240.

86 Seyit Höcük and others, *Economies of Scope in Data Aggregation: Evidence from Health Data* (TILEC Discussion Paper No 020, 2022, 10 November 2022) https://ssrn.com/abstract=4338447 or http://dx.doi.org/10.2139/ssrn.4338447.

87 *ibid*.

88 *ibid*.

89 Bertin Martens, ‘An Institutional Economics Approach to Data Spaces as Data Market Intermediaries’ [2024] SSRN Journal <https://www.ssrn.com/abstract=4731126>.

90 Martens, n79.

91 David Rubinfeld, ‘Data Portability and Interoperability: An EU–US Comparison’ (2024) 57 *European Journal of Law and Economics* 163 https://doi.org/10.1007/s10657-023-09781-w.

92 Directive (EU) 2019/1024 of the European Parliament and of the Council on open data and the re-use of public sector information [2019] OJ L172/56 (Open Data Directive).

93 Lundqvist, n64, 2.

94 C S Hutchinson, ‘Broad Collection of Consumer Data by Big Tech: Exclusionary or Exploitative Abuse?’ (2024) 20 European Competition Journal 610 https://doi.org/10.1080/17441056.2024.2340868.

95 DSSC, ‘Regulatory Compliance’ https://dssc.eu/space/BVE2/1071253931/Regulatory+Compliance.

96 European Union, ‘When Open Data Meets Data Spaces’, n14.

97 Martens and others, n79, 19-22.

98 AD4GD, ‘What Is the Green Deal Data Space?’ (AD4GD) https://ad4gd.eu/what-is-the-green-deal-data-space/.

99 Regulation (EU) 2016/679 of the European Parliament and of the Council on the protection of natural persons with regard to the processing of personal data (General Data Protection Regulation) [2016] OJ L119/1, art 20.

100 Martens and others,n79, 40–42, 47–48.

101 EU Commission, ‘Sustainable and Smart Mobility Strategy – Putting European Transport on Track for the Future’ (SWD 331 final) (2020) 16.

102 Data Spaces Support Centre, *Generative AI and Data Spaces* (White Paper, 2024) 25–27.

103 Philipp Hacker, Johann Cordes and Janina Rochon, ‘Regulating Gatekeeper Artificial Intelligence and Data: Transparency, Access and Fairness under the Digital Markets Act, the General Data Protection Regulation and Beyond’ (2023) *European Journal of Risk Regulation* 14(1) 63, 73–75.

104 Inge Graef, Martin Husovec and Nadezhda Purtova, ‘Data Portability and Data Control: Lessons for an Emerging Concept in EU Law’ (2018) 19 *German Law Journal* 1359, 1369–1370.

105 Green.Dat.AI Project, ‘Green.Dat.AI: A Data Spaces Architecture for Enhancing Green AI Services’ (2025) https://greendatai.eu.

106 Ioannis Chrysakis and others, ‘Multi-Partner Project: Green.Dat.AI: A Data Spaces Architecture for Enhancing Green AI Services’ (2025) *DATE Conference Proceedings* 1, 3–4.

107 *ibid*, 6-8.

108 *ibid*, 10-13.

109 Lundqvist, n64, 2.

110 Martens and others, n79.

111 Rubinfeld, n93, 170–72.

112 Heike Schweitzer, ‘The Art of Making Data Portability Effective: Lessons from European Competition Law’ (2019) 12 *Journal of European Competition Law & Practice* 5, 12–14.

113 DSSC, “Data Spaces’ Synergies” https://dssc.eu/space/DSSE/758350768/Data+Spaces'+Synergies.

114 DEPLOYTOUR Project, ‘Deployment of a Common European Tourism Data Space (ETDS)’ https://deploytour.eu.

115 European Commission, ‘European Mobility Data Space’,n 62.

116 European Commission, ‘European Health Data Space’, n61.

117 European Commission, ‘Public Procurement Data Space (PPDS)’ https://single-market-economy.ec.europa.eu/single-market/public-procurement/digital-procurement/public-procurement-data-space-ppds_en.

118 Otto and others, n5, 18.

119 R Raab and others, ‘Federated Electronic Health Records for the European Health Data Space’ (2023) 5 *The Lancet Digital Health* e840–e847 https://doi.org/10.1016/S2589-7500(23)00156-5.

120 Nikolaus Forgó and others, ‘Privacy-Preserving Techniques for Trustworthy Data Sharing’ in Edward Curry (ed), *Data Spaces: Design, Deployment and Future Directions* (Springer 2022) 331–333; D Hurtado Ramírez and others, *Technological Enablers for Privacy Preserving Data Sharing and Analysis* (Publications Office of the European Union, Luxembourg 2023) JRC134350 https://doi.org/10.2760/427718.

121 Luigi Coppolino and others, ‘Exploiting Data Spaces to Enable Privacy-Preserving Data Exchange in the Energy Supply Chain’ (ITASEC 2024) 6–7.

122 Forgó and others, n122, 333–336; European Commission, *Technological Enablers for Privacy Preserving Data Sharing and Analysis*, n122, 22–24.

123 European Commission, ‘Interoperability of Data Spaces: Building Europe’s Digital Future’ (Data.europa.eu, 27 February 2024) https://data.europa.eu/en/news-events/news/interoperability-data-spaces-building-europes-digital-future.

124 Proposal for a Regulation of the European Parliament and of the Council on the European Health Data Space COM (2022) 197 final.

125 *ibid*.

126 Case C-418/01 *IMS Health GmbH & Co OHG v NDC Health GmbH & Co KG* [2004] ECR I-5039.

127 Case T-210/04 *Microsoft Corp v Commission* ECLI:EU:T:2007:289, [2007] ECR II-3601.

128 DSSC, *Data Exchange* (Blueprint v2.0, 7 March 2025) https://dssc.eu/space/BVE2/1071255453/Data+Exchange.

129 BearingPoint, “Data Sovereignty: The Driving Force Behind Europe’s Sovereign Cloud Strategy” (BearingPoint Belgium) https://www.bearingpoint.com/en-be/insights-events/insights/data-sovereignty-the-driving-force-behind-europes-sovereign-cloud-strategy/.

130 *ibid*.

131 European Data Portal, *European Data Spaces and the Role of data.europa.eu* (Publications Office of the European Union 2023) 13–21 https://data.europa.eu/sites/default/files/report/Data_Spaces_Panel_Report_EN.pdf.

132 S Scerri, T Tuikka, I L de Vallejo and E Curry, ‘Common European Data Spaces: Challenges and Opportunities’ in E Curry, S Scerri and T Tuikka (eds), *Data Spaces* (Springer, Cham 2022) https://doi.org/10.1007/978-3-030-98636-0_16.

133 Bundeskartellamt, *Catena-X* (B6-22/22, 2022) paras 25–30.

134 Gaia-X, *Gaia-X Architecture Document – 22.10 Release: Overview* (Technical Committee, section 2.2) https://docs.gaia-x.eu/technical-committee/architecture-document/22.10/overview/.

135 Riccardo Albertoni, David Browning, Simon Cox, Alejandra N. Gonzalez-Beltran, Andrea Perego & Peter Winstanley, “The W3C Data Catalog Vocabulary, Version 2: Rationale, Design Principles, and Uptake” (arXiv, 15 March 2023) https://arxiv.org/pdf/2303.08883.

136 DSSC, *‘8 – Technology’* (DSSC Starter Kit, 1 November 2024) https://dssc.eu/space/SK/812515362/8+-+Technology.

137 Google and Alphabet v European Commission (Google Shopping) Case T-612/17 EU:T:2021:763.

138 European Commission, *Comfort Letter to Gaia-X* (2021) 3–4.

139 Alexandre de Corniere, Greg Taylor, *Data and Competition: A Simple Framework* (Toulouse School of Economics), Working Paper No 1404, August 2024.

140 A Duisberg, ‘Legal Aspects of IDS: Data Sovereignty — What Does It Imply?’ in Boris Otto, Michael ten Hompel and Stefan Wrobel (eds), *Designing Data Spaces: The Ecosystem Approach to Competitive Advantage* (Springer 2022) ch 5, 61–63 https://doi.org/10.1007/978-3-030-93975-5_5.

141 *ibid*, 63.

142 *ibid*.

143 Scerri et al, n134.

144 Tobias Dam, Andreas Krimbacher and Sebastian Neumaier, ‘Policy Patterns for Usage Control in Data Spaces’ (2023) arXiv 1–3 https://arxiv.org/pdf/2309.11289.

145 Regulation (EU) 2022/868 of the European Parliament and of the Council of 30 May 2022 on European data governance (Data Governance Act) [2022] OJ L152/1, ch 3.

146 *ibid*, recitals 25–27.

147 Simon Vande Walle, *Data Spaces, Data Ecosystems and Competition Law: A Spotlight on the EU’s Gaia-X, Catena-X and Cofinity-X Projects* (2023) https://ssrn.com/abstract=4732291.

148 European Commission, ‘Open Data’ (Digital Strategy) https://digital-strategy.ec.europa.eu/en/policies/open-data.

149 Open Data Directive, n94.

150 European Commission, *A European Strategy for Data*, n2.

151 DSSC, *Data Spaces Blueprint v2.0 – Home*https://dssc.eu/space/BVE2/1071251457/Data+Spaces+Blueprint+v2.0+-+Home

152 Data Governance Act, art 29.

153 European Commission, *Comfort Letter to Gaia-X* (2021) 5–6.

154 Radio Telefis Eireann (RTE) and Independent Television Publications Ltd (ITP) v Commission of the European Communities (Joined Cases C-241/91 P and C-242/91 P) [1995] ECR I-743 (CJEU).

155 IMS Health GmbH & Co OHG v NDC Health GmbH & Co KG (Case C-418/01) [2004] ECR I-5039 (CJEU).

156 Martens, n91.

157 *ibid*.

158 Jens Prüfer and Christoph Schottmüller, *Competing with Big Data* (2017) TILEC Discussion Paper No 2017-006, SSRN https://ssrn.com/abstract=2918726

159 *ibid*, 3.

160 Prüfer and Schottmüller, n160 , 970–971, 989–992.

161 Corniere, Taylor, n141.

162 European Commission, *Staff Working Document on* Common European *Data Spaces* SWD(2022) 45 final, (Brussels, 23 February 2022) 31.

163 Bundeskartellamt, *Decision on Catena-X* (2022) paras 25–30.

164 CADE, *Administrative Proceeding No. 08700.004431/2021-13 – Catena-X* (2023).

165 H Schweitzer and R Welker, ‘A Legal Framework for Access to Data – a Competition Policy Perspective’ in J Drexl (ed), *Data Access, Consumer Interests and Public Welfare* (Nomos 2021) 148, n 154.

166 Directive (EU) 2022/2464 as regards corporate sustainability reporting OJ L322/15 (CSRD); Directive (EU) 2024/1760 on corporate sustainability due diligence OJ L, 2024/1760 (CSDDD).

167 König, n1, 497.

## References

[1] AD4GD, “What Is the Green Deal Data Space?” (AD4GD) https://ad4gd.eu/what-is-the-green-deal-data-space/.

[2] Riccardo Albertoni and others, ‘The W3C Data Catalog Vocabulary, Version 2: Rationale, Design Principles, and Uptake’ (2024) 6(2) Data Intelligence 457-487, doi:10.1162/dint_a_00241.

[3] AWS Data Exchange (Amazon Web Services), “AWS data exchange” https://aws.amazon.com/data-exchange/.

[4] Azure Marketplace (Microsoft), “Azure marketplace” https://azuremarketplace.microsoft.com.

[5] Bacco M. (2024). What are data spaces? Systematic survey and future outlook. Data Brief.

[6] BearingPoint, “Data sovereignty: the driving force behind europe’s sovereign cloud strategy” (2023) https://www.bearingpoint.com/en/our-success/insights/data-sovereignty-the-driving-force-behind-europes-sovereign-cloud-strategy/.

[7] Beyvers F and others, ‘Towards FAIR and federated data ecosystems for interdisciplinary research’ (2025) arXiv:2504.20298 https://arxiv.org/abs/2504.20298. 10.1371/journal.pcbi.1013806 PMC12904567 41686858

[8] Borowicz M.K. (2025). Competition, standards, and EU data spaces. Data Brief.

[9] Bundeskartellamt, ‘Catena-X automotive network’ (Decision B6-22/22, 2022).

[10] CADE, Administrative proceeding no. 08700.004431/2021-13 — Catena-X (2023).

[11] Case AT.40462 *amazon marketplace* C(2022) 9442 final.

[12] Case C-418/01 *IMS health GmbH & Co OHG v NDC health GmbH & Co KG* [2004]ECR I-5039.Case C-241/91 P and C-242/91 P *radio telefis eireann (RTE) and independent television publications Ltd (ITP) v Commission* [1995]ECR I-743.

[13] Case C-252/21 *meta platforms inc and others v bundeskartellamt* eu:C:2023:537.

[14] Case C-7/97 *oscar bronner GmbH & Co KG v mediaprint zeitungs- und zeitschriftenverlag GmbH & Co KG* [1998]ECR I-7791.

[15] Case T-201/04 *microsoft corp v commission* [2007]ECR II-3601.

[16] Case T-612/17 *Google LLC and alphabet inc v commission (Google Shopping)* EU:T:2021:763.

[17] Centre of Excellence for Data Sharing & Cloud, “SCSN and Catena-X: how to facilitate interoperability between data spaces” https://coe-dsc.nl/scsn-and-catena-x-how-to-facilitate-interoperability-between-data-spaces/.

[18] Chrysakis I. (2025). DATE Conference Proceedings.

[19] Communication from the Commission, *Guidelines on the applicability of Article 101 of the Treaty on the Functioning of the European Union to horizontal cooperation agreements* [2023]OJ C 259/1.Consolidated version of the treaty on the functioning of the European Union [2016]OJ C 202/47.

[20] Coppolino L and others, ‘Exploiting data spaces to enable privacy preserving data exchange in the energy supply Chain’ (ITASEC 2024).

[21] Corniere A de, Taylor G, Data and competition: a simple framework (toulouse school of economics, working paper no 1404, August 2024)

[22] Crémer J, de Montjoye YA and Schweitzer H, Competition policy for the digital era: final report (european commission, publications office of the European Union 2019) 44–55, doi:10.2763/407537.

[23] Custers B., Malgieri G. (2022). Priceless data: why the EU fundamental right to data protection makes data ownership unsustainable. Comput. Law Secur. Rev..

[24] D’Acquisto G. and others, “European digital identity wallets: compliance and interoperability” (ENISA 2023).

[25] Dam T, Krimbacher A and Neumaier S, “Policy patterns for usage control in data spaces” (2023) arXiv 1–3 https://arxiv.org/pdf/2309.11289.

[26] Data Spaces Support Centre, “Generative AI and data spaces” (White Paper, 2024) 25–27.

[27] DEPLOYTOUR Project, “Deployment of a common european tourism data space (ETDS)” https://deploytour.eu.

[28] Directive (EU) 2019/1024 of the European parliament and of the council of 20 June 2019 on open data and the re-use of public sector information [2019]OJ L 172/56.

[29] Directive (EU) 2022/2464 as regards corporate sustainability reporting [2022]OJ L 322/15.

[30] Directive (EU) 2024/1760 on corporate sustainability due diligence [2024]OJ L 1760/1.

[31] DSSC (Data Space Support Centre), “Glossary: 1.3 Definition of data space and definition of synergy” (24 October 2024) https://dssc.eu/space/DSSE/791740421/1.3+Definition+of+data+space+and+definition+of+synergy.

[32] DSSC, ‘8 – Technology’ (DSSC Starter Kit, 1 November 2024) https://dssc.eu/space/SK/812515362/8+-+Technology.

[33] DSSC, “Data spaces’ synergies” https://dssc.eu/space/DSSE/758350768/Data+Spaces'+Synergies.

[34] DSSC, “Governance building blocks” (7 March 2025) https://dssc.eu/space/BVE2/1071253634/Governance+Building+Blocks.

[35] DSSC, “Introduction — Key concepts of data spaces” https://dssc.eu/space/BVE2/1071251613/Introduction%2B-%2BKey%2BConcepts%2Bof%2BData%2BSpaces.

[36] DSSC, “Regulatory compliance” https://dssc.eu/space/BVE2/1071253931/Regulatory+Compliance.

[37] DSSC, Data exchange (Blueprint v2.0, 7 March 2025) https://dssc.eu/space/BVE2/1071255453/Data+Exchange.

[38] DSSC, Data space design principles: version 2.0 (September 2024) https://dssc.eu/download/attachments/766181377/Data%20Space%20Design%20Principles.pdf.

[39] DSSC, Data spaces blueprint v2.0 – home https://dssc.eu/space/BVE2/1071251457/Data+Spaces+Blueprint+v2.0+-+Home.

[40] Duisberg A., Otto B., ten Hompel M., Wrobel S. (2022). Designing Data Spaces.

[41] European Commission, “Data governance act explained” (Shaping Europe’s Digital Future, 11 October 2024) https://digital-strategy.ec.europa.eu/en/policies/data-governance-act-explained.

[42] European Commission, “European health data space regulation (EHDS)” https://health.ec.europa.eu/ehealth-digital-health-and-care/european-health-data-space-regulation-ehds_en.

[43] European Commission, “European mobility data space” (2022) https://ec.europa.eu/info/law/better-regulation/have-your-say/initiatives/13566-Transport-data-creating-a-common-European-mobility-data-space-communication-_en.

[44] European Commission, A European strategy for data COM (2020) 66 final, 19 February 2020, https://eur-lex.europa.eu/legal-content/EN/TXT/?uri=CELEX%3A52020DC0066.

[45] European Commission, Comfort letter to Gaia-X (2021)

[46] European Commission, Common European Data Spaces (Shaping Europe’s Digital Future, 9 July 2025) https://digital-strategy.ec.europa.eu/en/policies/data-spaces.

[47] European Commission, “Open data” (Digital Strategy) https://digital-strategy.ec.europa.eu/en/policies/open-data.

[48] European Commission, “Public procurement data space (PPDS)” https://single-market-economy.ec.europa.eu/single-market/public-procurement/digital-procurement/public-procurement-data-space-ppds_en.

[49] European Commission, Staff working document on common european data spaces SWD(2022) 45 final (Brussels, 23 February 2022).

[50] European Commission, Sustainable and smart mobility strategy – Putting European transport on track for the future SWD 331 final (2020) 16.

[51] European Commission (2023).

[52] European Data Portal, “European data spaces and the role of data.europa.eu” (Publications Office of the European Union, 2023) 13–21 https://data.europa.eu/sites/default/files/report/Data_Spaces_Panel_Report_EN.pdf

[53] European Parliament and Council, Data Governance Act (Regulation (EU) 2022/868) [2022]OJ L 152, recital 2.

[54] European Union, “When open data meets data spaces” (data.europa.eu, 2022) https://data.europa.eu/en/publications/datastories/when-open-data-meets-data-spaces.

[55] Forgó N., Curry E. (2022). Data Spaces: Design, Deployment and Future Directions.

[56] Gaia-X, “Gaia-X architecture document –22.10 Release: overview (Technical Committee, section 2.2)” https://docs.gaia-x.eu/technical-committee/architecture-document/22.10/overview/.

[57] García-Morales E. (2024). The European health data space: governance and access. Health Policy.

[58] Gieß S., Schoormann T., Möller F., Heisel M., Taveter L., Felfernig A. (2025). Perspectives in Business Informatics Research.

[59] Google and Alphabet v European Commission (Google Shopping) Case T-612/17 EU:T:2021:763

[60] Google cloud public datasets (Google Cloud), “Google cloud public datasets” https://cloud.google.com/datasets.

[61] Graef I., Prüfer J. (2021). Governance of data sharing: a law & economics proposal. Res. Policy.

[62] Graef I., Husovec M., Purtova N. (2018). Data portability and data control: lessons for an emerging concept in EU Law. Germ. Law J..

[63] Green.Dat.AI Project, “A data spaces architecture for enhancing green AI services” (2025) https://greendatai.eu.

[64] Hacker P., Cordes J., Rochon J. (2023). Regulating gatekeeper artificial intelligence and data: transparency, access and fairness under the digital markets act, the general data protection regulation and beyond. Europ. J. Risk Regul..

[65] Hauff M. (2024). CEUR Workshop Proceedings.

[66] Höcük S and others, Economies of scope in data aggregation: evidence from health data (TILEC Discussion Paper No 020, 2022) https://ssrn.com/abstract=4338447.

[67] Hutchinson C.S. (2024). Broad collection of consumer data by big tech: exclusionary or exploitative abuse?. Europ. Compet. J..

[68] International Data Spaces Association (2021). https://www.internationaldataspaces.org/.

[69] Jurmu M. (2023). Exploring the role of federated data spaces in implementing twin transition within manufacturing ecosystems. Sensors.

[70] Kerber W., Schweitzer H. (2017). Interoperability in the digital economy. Jipitec.

[71] König P.D. (2022). Fortress Europe 4.0? an analysis of EU data governance through the lens of the resource regime concept. Euro. Policy Analy..

[72] Lundqvist B. (2023). An access and transfer right to data—from a competition law perspective. J. Antitrust Enforcem..

[73] Lundqvist B. (2020). Data access and portability and EU competition law. CPI Antit. Chron..

[74] Martens B. (2020). JRC Digital Economy Working Paper 2020-05, European Commission.

[75] Martens B. (2024). An institutional economics approach to data spaces as data market intermediaries. SSRN J..

[76] Murati E. (2025). Overlooking digital collusion risks in the EU’s agenda for a single European data space(s). Europ. Compet. J..

[77] Nagel L and Lycklama D, “Design principles for data spaces” (Zenodo, 2021) 76–79 https://zenodo.org/record/5244997.

[78] National Institute of Standards and Technology (NIST), NIST big data interoperability framework: volume 1, definitions (Special Publication 1500-1r2, 2019) 11, 16, 18, 23, 31–32 10.6028/NIST.SP.1500-1r2.

[79] OECD, Going digital to advance data governance for growth and well-being (OECD Publishing 2022) 8–10, 30, 32–34 10.1787/e3d783b0-en.

[80] Otto B., ten Hompel M., Wrobel S. (2022). https://link.springer.com/10.1007/978-3-030-93975-5.

[81] Proposal for a regulation of the European parliament and of the council on the European Health Data Space COM (2022) 197 final

[82] Prüfer J., Schottmüller C. (2017). TILEC Discussion Paper No 2017-006, SSRN.

[83] Raab R. (2023). Federated electronic health records for the European health data space. Lancet Dig. Health.

[84] Radio Telefis Eireann (RTE) and independent television publications Ltd (ITP) v commission (Joined Cases C-241/91 P and C-242/91 P) ECR I-743

[85] Regulation (EU) 2016/679 of the European parliament and of the council on the protection of natural persons with regard to the processing of personal data and on the free movement of such data (General Data Protection Regulation) [2016]OJ L119/1.

[86] Regulation (EU) 2022/868 of the European parliament and of the council of 30 May 2022 on European data governance (Data Governance Act) [2022]OJ L 152/1.

[87] Regulation (EU) 2023/2854 of the European parliament and of the council of 13 December 2023 on harmonised rules on fair access to and use of data (Data Act) [2023]OJ L 2854/1.

[88] Regulation (EU) 2022/1925 of the European parliament and of the council of 14 September 2022 on contestable and fair markets in the digital sector (Digital Markets Act) [2022]OJ L 265/1.

[89] Rubinfeld D. (2024). Data portability and interoperability: an EU–US comparison. Euro. J. Law Econom..

[90] Scerri S., Tuikka T., de Vallejo I.L., Curry E., Curry E., Scerri S., Tuikka T. (2022). Data Spaces.

[91] Schöppenthau F. (2023). Building a digital manufacturing as a service ecosystem for catena-X. Sensors.

[92] Schweitzer H. (2019). The art of making data portability effective: lessons from European competition law. J. Europ. Compet. Law Pract..

[93] Schweitzer H., Welker R., Drexl J. (2021). Data Access, Consumer Interests and Public Welfare.

[94] SCSN (Smart Connected Supplier Network), “SCSN – Smart Connected Supplier Network” (May 2024) https://manufacturingdataspace-csa.eu/wp-content/uploads/2024/05/SCSN-Smart-Connected-Supplier-Network.pdf.

[95] Serrano M and others, “Data space best practices for data interoperability in FinTechs” in Connecting Data Spaces and Data Marketplaces: A Comprehensive Approach to Build Trust in Data Sharing (2022) 252–253.

[96] Statista, “Volume of data created, captured, copied, and consumed worldwide from 2010 to 2023, with forecasts from 2024 to 2028” https://www.statista.com/statistics/871513/worldwide-data-created.

[97] Steinbuss S. (2025).

[98] Steiner B., Münch C. (2024). Leveraging digital data spaces in purchasing and supply management: paving the way to the circular economy exemplified by Catena-X. J. Purchas. Supp. Manage..

[99] Tardieu H., Otto B. (2022). Designing Data Spaces.

[100] Vande Walle S. (2023). Data spaces, data ecosystems and competition law: a spotlight on the EU’s Gaia-X, Catena-X and Cofinity-X projects. SSRN Elec. J..

[101] Wilkinson M.D. (2016). The FAIR guiding principles for scientific data management and stewardship. Scient. Data.

[102] Zappa C and others, Connecting data spaces and data marketplaces: a comprehensive approach to build trust in data sharing (2022) 134-135.

[103] Zingales N., Kokkoris I. (2021). Research Handbook in Competition Enforcement.

[104] Zoboli L., Bernatt M., Zoboli L., Bernatt M. (2025). Data Sharing Regulation in Europe.

